# Past Sodium Intake, Contemporary Sodium Intake, and Cardiometabolic Health in Southwest Coastal Bangladesh

**DOI:** 10.1161/JAHA.119.014978

**Published:** 2020-09-02

**Authors:** Abu Mohd Naser, Mahbubur Rahman, Leanne Unicomb, Solaiman Doza, Shahjada Selim, Monjila Chaity, Stephen P. Luby, Shuchi Anand, Lisa Staimez, Thomas F. Clasen, Unjali P. Gujral, Matthew O. Gribble, K. M. Venkat Narayan

**Affiliations:** ^1^ Emory Global Diabetes Research Center Hubert Department of Global Health Rollins School of Public Health Emory University Atlanta GA; ^2^ International Centre for Diarrhoeal Disease Research Bangladesh (icddr,b) Dhaka Bangladesh; ^3^ Department of Endocrinology Bangabandhu Sheikh Mujib Medical University Dhaka Bangladesh; ^4^ Carle Foundation Hospital Urbana IL; ^5^ Division of Infectious Diseases and Geographic Medicine Stanford University Stanford CA; ^6^ Division of Nephrology School of Medicine Stanford University Stanford CA; ^7^ Gangarosa Department of Environmental Health Sciences Rollins School of Public Health Emory University Atlanta GA; ^8^ Department of Epidemiology Rollins School of Public Health Emory University Atlanta GA

**Keywords:** 24‐hour urine sodium, cardiometabolic biomarkers, diabetes mellitus, metabolic syndrome, proteinuria, sodium intake, urine sodium, Diet and Nutrition, Epidemiology, Cardiovascular Disease, Obesity

## Abstract

**Background:**

We compared the relationship of past and contemporary sodium (Na) intake with cardiometabolic biomarkers.

**Methods and Results:**

A total of 1191 participants' data from a randomized controlled trial in coastal Bangladesh were analyzed. Participants provided 24‐hour urine Na (24UNa) data for 5 monthly visits. Their fasting blood glucose, total cholesterol, triglycerides, high‐density lipoprotein, blood pressure, and 24‐hour urine protein were measured at the fifth visit. Participants' mean 24UNa over the first 4 visits was the past Na, and 24UNa of the fifth visit was the contemporary Na intake. We estimated the prevalence ratios of elevated cardiometabolic biomarkers and metabolic syndrome across 24UNa tertiles by multilevel logistic regression using participant‐, household‐, and community‐level random intercepts. Models were adjusted for age, sex, body mass index, smoking, physical activity, alcohol consumption, sleep hours, religion, and household wealth. Compared with participants in tertile 1 of past urine Na, those in tertile 3 had 1.46 (95% CI, 1.08–1.99) times higher prevalence of prediabetes or diabetes mellitus, 5.49 (95% CI, 2.73–11.01) times higher prevalence of large waist circumference, and 1.60 (95% CI, 1.04–2.46) times higher prevalence of metabolic syndrome. Compared with participants in tertile 1 of contemporary urine Na, those in tertile 3 had 1.93 (95% CI, 1.24–3.00) times higher prevalence of prediabetes or diabetes mellitus, 3.14 (95% CI, 1.45–6.83) times higher prevalence of proteinuria, and 2.23 (95% CI, 1.34–3.71) times higher prevalence of large waist circumference.

**Conclusions:**

Both past and contemporary Na intakes were associated with higher cardiometabolic disease risk.

**Nonstandard Abbreviations and Acronyms**
AHA: American Heart Association
DASH: Dietary Approaches to Stop Hypertension
RCS: restricted cubic spline

**Clinical Perspective**

**What Is New?**

- Both past and contemporary sodium (Na) intakes are associated with high fasting blood glucose and urine protein excretion.
- Past Na intake is associated with a high prevalence of prediabetes or diabetes mellitus, proteinuria, large waist circumference, and metabolic syndrome.
- Contemporary Na intake is associated with a high prevalence of prediabetes or diabetes mellitus, proteinuria, and large waist circumference.

**What Are the Clinical Implications?**

- Reduction in Na intake will benefit patients with prediabetes and diabetes mellitus, high urine protein excretion, and metabolic syndrome.

High dietary sodium (Na) intake is the leading dietary risk for death and disability.^1^ Most epidemiologic studies with robust measurement of Na intake suggest that high Na intake increases tapphe risks of hypertension and cardiovascular diseases.^2^, ^3^, ^4^, ^5^ Therefore, population‐level reductions in Na intake are priority interventions for reducing cardiovascular diseases.^6^ Multiple complex and interconnected physiologic mechanisms are linked with high Na intake and cardiometabolic diseases, including fluid homeostasis, hormonal, neuronal, inflammatory, and immune mechanisms.^5^ Studies support that even in the absence of an increase in blood pressure (BP), high Na intake can adversely affect target organs, including the blood vessels, heart, kidneys, and brain.^7^

An average of 93% of ingested daily Na is excreted in 24‐hour urine.^8^ Therefore, salt loading–associated hemodynamic changes can be better evaluated by measuring health outcomes within 24 hours of salt load. Nevertheless, some health outcomes (eg, arterial stiffness, body fat deposition, chronic kidney disease, left ventricular hypertrophy) could be more related to retrospective Na intake than the contemporary Na intake.^5^, ^9^, ^10^ Metabolic disease such as type 2 diabetes mellitus is a significant public health burden in many Asian communities, including South Asians.^11^ High Na intake can influence type 2 diabetes mellitus through a number of pathways, including increasing the adipose tissue mass, leptin production, and enhancing insulin sensitivity (Figure S1).^12^, ^13^ Such biochemical cascades of enhancing insulin sensitivity may require weeks to be activated following Na intake. Because of the rhythmic hormonal influence of urine aldosterone and cortisol, total body Na content also exhibits a longer‐term rhythm.^14^ Hence, it is likely that many cardiometabolic parameters are influenced by retrospective Na intake.

Appropriate measurement of Na intake^15^ and studying the pleiotropic effects of high Na intake on different cardiometabolic pathways^16^ can better inform the public health burden of high Na intake. Currently, limited data exist on the retrospective or past Na exposure and cardiometabolic biomarkers' relationships and how such relationships differ from the contemporary Na exposure. We measured the past Na exposure of a population by averaging 24‐hour urine Na (24UNa) collected over months to evaluate its associations with cardiometabolic biomarkers measured prospectively, including fasting plasma glucose, total cholesterol, triglycerides, high‐density lipoprotein cholesterol (HDL‐C), uric acid, 24‐hour urine total protein, and metabolic syndrome.

## Methods

### Data Source and Study Setting

**Figure 1:**
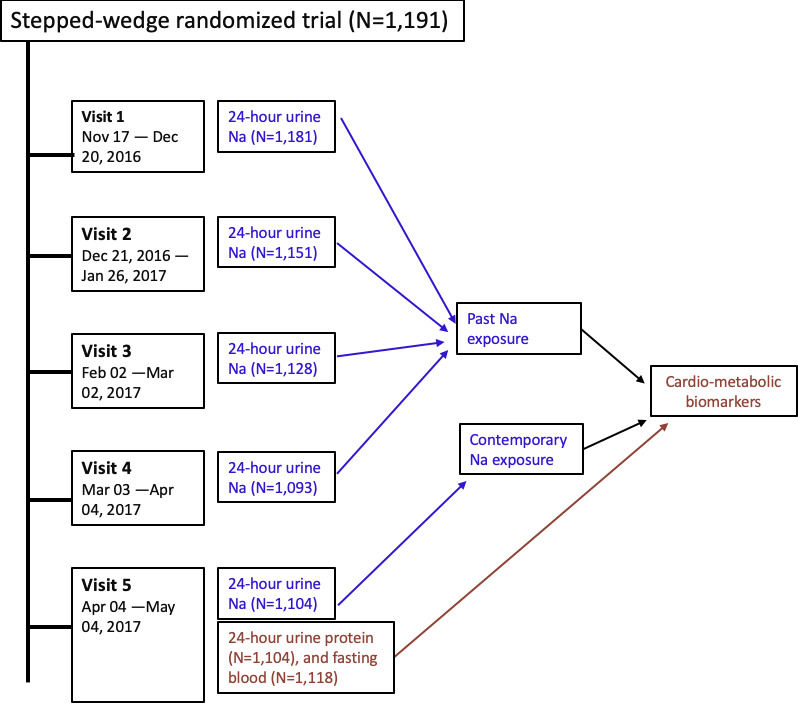
Data sources for analyses.

The data that support the findings of this study are available from the corresponding author upon reasonable request. We analyzed data from a stepped‐wedge randomized controlled trial (NCT02746003) conducted in 16 communities in southwest coastal Bangladesh led by the International Centre for Diarrhoeal Disease Research, Bangladesh (icddr,b).^17^, ^18^ Stepped‐wedge trial is a design where random and sequential crossover of clusters or communities occur from control to intervention arms until all clusters get the intervention.^19^ Therefore, more clusters are enrolled to the intervention arms at the end stage of the trial than the early stages.^19^ The study areas are affected by seawater intrusion, and the groundwater aquifers in the region contain saline water.^20^ Communities have high Na intake through drinking water during the dry seasons and their Na intakes varies when drinking water salinity changes.^21^ The stepped‐wedge trial evaluated the health impacts of providing access to managed aquifer recharge,^17^ a hydrologic intervention to lower aquifer salinity, during the dry season of December 2016 to April 2017, when water salinity was high. Hence, participants had varying levels of Na intake during the course of the study. We followed up 1191 participants from 542 households at 5 monthly time points. During each of the 5 visits, we collected participants' drinking water salinity data and 24‐hour urine samples. In the final (fifth) visit, we measured BP and 24‐hour urine total protein and collected fasting blood to measure plasma glucose, cholesterol, triglycerides, HDL‐C, and uric acid (Figure 1).

### Cardiometabolic Disease Risk Factor Data

We collected the demographics (age, sex, religion) and anthropometric (height, weight, and waist circumference) characteristics of the participants and socioeconomic information (eg, household asset). We also collected data on smoking, work‐related physical exercise, alcohol consumption, sleep hours, households' use of table salt for cooking, and participants' consumption of additional table salt with food. Self‐reported information about hypertension, diabetes mellitus, and chronic kidney disease were collected.

### Urine Sample Collection and Na and Protein Measurement

During each visit, participants received a 4‐L plastic container for 24‐hour urine collection and a plastic mug to transfer the voided urine to the 4‐L plastic container. Participants were instructed to discard their first‐morning urine and to begin the 24‐hour urine collection by transferring the second morning void of the day and then to transfer all voids of the day and night including the next morning's first void to the 4‐L plastic container. The total volume of 24‐hour collected urine was recorded, and a 15 mL sample was taken from the 4‐L plastic container after stirring. All urine samples were transported to a field laboratory at 2 to 8°C for processing and analysis on the same day. Direct ion‐selective electrode method^22^ was used for urine Na measurements using a semiautomatic electrolyte analyzer (Biolyte2000, Bio‐care Corporation, Taiwan; coefficient of variation [CV], ±5%); colorimetric method was used for urine total protein using a semiautomatic biochemistry analyzer (Evolution 3000, BSI, Italy; CV, <1%); Jaffe reaction was used for urine creatinine measurement.^23^

### Defining Past and Contemporary Na Exposure

We considered the mean of 24UNa of the first 4 visits as the measure of past Na exposure. Of the 1191 participants, 1025 had no missing 24‐hour urine samples in any of the first 4 visits, 135 had missing sample in one visit, 18 had missing samples in 2 visits, 12 had missing samples in 3 visits, and 4 did not have any urine samples. Participants who had no missing urine samples or had 1 missing samples in the first 4 visits were used to calculate past Na exposure (n=1160; Figure 1). We considered 24UNa of the fifth visit was as the contemporary Na exposure (n=1104; Figure 1). Therefore, of the 1191 participants enrolled in the trial, 97.4% had past Na exposure and 92.7% had contemporary Na exposure.

### Fasting Blood Collection, Cardiometabolic Biomarkers, and BP Measurement

Trained phlebotomists collected 5 mL of fasting blood by venipuncture using aseptic precautions. Blood samples were transferred to a field laboratory centrifugation at 894*g* relative centrifugal force for 15 minutes at ambient temperature for plasma separation, and then aliquots were stored in a −20°C freezer. Blood glucose was measured by hexokinase method^24^, total cholesterol and uric acid were measured by enzymatic endpoint method^25^, HDL‐C was measured by direct clearance method^26^, and triglycerides were measured by enzymatic colorimetric method.^27^ The semiautomatic biochemistry analyzer (Evolution 3000, BSI, Italy; CV, <1%) was used to analyze all cardiometabolic biomarkers.

Participants' BP was measured at their homestead using Omron HEM‐907 (accuracy, within ±4 mm Hg; Kyoto, Japan) digital BP monitors between 7.30 am and 2.00 pm. An appropriately sized cuff was used based on mid–upper arm circumference. BP was measured 3 times. The arithmetic mean of 3 measurements was used in analyses.

The American Heart Association (AHA) criteria were used to define elevated triglycerides (≥150 mg/dL), elevated fasting glucose (≥5.5 mmol/L labeled as prediabetes or diabetes mellitus), and reduced HDL‐C (<40 mg/dL for male; <50 mg/dL for female).^28^ Plasma cholesterol was considered elevated when ≥200 mg/dL for both sexes, uric acid was considered elevated if ≥7 mg/dL for males and ≥6 mg/dL for females, and proteinuria was considered when urine total protein was ≥300 mg/dL for both sexes.^29^ We defined metabolic syndrome using the AHA criteria of ≥3 of the following criteria: triglycerides ≥150 mg/dL; fasting glucose ≥5.5 mmol/L, HDL‐C <40 mg/dL for male or <50 mg/dL for female, systolic BP ≥130 or diastolic BP ≥85, and waist circumference ≥92 cm for male or ≥89 cm for female.^28^

### Statistical Analysis

We determined the proportion of variables and mean of all biomarkers across tertiles of Na exposure. We used the 2‐sample test of proportions or t test, as applicable, to compare the proportions or means with respect to reference tertile. Body mass index (BMI) was categorized according to WHO Asian cut points: underweight (BMI, <18.5 kg/m^2^), normal weight (BMI, 18.5 to <23 kg/m^2^), overweight (BMI, 23.0 to <27.5 kg/m^2^), and obese (BMI, ≥27.5 kg/m^2^).^30^ Household wealth quintiles were calculated from household wealth scores using principal component analysis of household asset data including ownership of a refrigerator, television, mobile phone, motorcycle, bicycle, sewing machine, chair, table, wristwatch, wardrobe, wooden cot, motor pump, rice husking machine, motorized rickshaw, car, and access to electricity.

To assess the nonlinear relationship between 24UNa and each of the cardiometabolic biomarkers, we first plotted the restricted cubic spline (RCS) plots to visually assess the nature of the relationship and to detect any nonlinearity.^31^ We used a default of 4 knots placed at symmetrical percentiles (fifth, 35th, 65th, and 95th) of past and contemporary Na to create flexible smooth plots. RCS plots assume cubic polynomials in segments after the first knot and before the last knot.^31^ Hence, our spline plots could identify a nonlinear relationship between the 24UNa and cardiometabolic biomarkers between fifth and 95th percentile distribution of 24UNa measurements. We used multilevel linear models with random intercepts for households and communities to adjust clustering at household and community levels. RCS plots were adjusted for age, sex, BMI, smoking, physical activity, alcohol consumption, sleep hour categories, religion, and household wealth. We used the Wald test for detecting departure from linearity after running the model.^31^

We then determined difference in mean biomarkers across tertiles of past and contemporary Na exposure using the similar multilevel linear models. Urine total protein had skewed distributions. Hence, we used multilevel gamma regression models^32^ to estimate the ratio of median urine total protein between Na tertiles. We also determined the prevalence ratios of elevated cardiometabolic biomarkers and metabolic syndrome for participants in tertile 2 and tertile 3 of 24UNa using multilevel logistic regression models considering tertile 1 as the reference group.

All multilevel models included 2‐level random intercepts to account for clustering of participants within households and households within communities. We estimated the models using maximum likelihood and reported cluster robust standard errors. We sequentially reported findings from unadjusted models; models adjusted for age, sex, and BMI; and models that additionally adjusted for smoking, physical activity, alcohol consumption, sleep hour categories, religion, and household wealth. We included age and BMI as a continuous variable in the models, but other covariates were included as categorical variables. Categories for all covariates are described in Table 1.

**Table 1:** Characteristics of the Study Participants and Cardiometabolic Biomarkers Across Tertile of Past Na Exposure

Characteristics	Tertile 1 % (n) or Mean (SD)	Tertile 2 % (n) or Mean (SD) [*P* Value*]	Tertile 3 % (n) or Mean (SD) [*P* Value*]
Age category, % (N)			
20 to <40 y (n=528)	30.1 (159)	34.9 (184) [0.344]	35.0 (185) [0.334]
40 to <60 y (n=426)	32.6 ( 139)	32.2 (137) [0.943]	35.2 (150) [0.641]
≥60 y (n=169)	46.2 (78)	30.2 (51) [0.070]	23.7 (40) [0.018]
Sex, % (N)			
Female (n=673)	33.8 (152)	34.7 ( 156) [0.868]	31.6 (142) [0.688]
Male (n=450)	33.3 (224)	32.1 (216) [0.789]	34.6 (233) [0.769]
BMI categories, % (n)			
Underweight (n=183)	44.8 (82)	33.9 (62) [0.186]	21.3 (390) [<0.001]
Normal weight (n=693)	36.1 (250)	32.8 (227) [0.449]	31.2 (216) [0.265]
Overweight ( n=198)	17.7 (35)	34.2 (68) [0.079]	47.1 (95) [0.002]
Obese (n=33)	12.1 (4)	30.3 (10) [0.478]	57.6 (19) [0.098]
Smoker, % (n)			
Never (n=575)	32.0 (184)	31.8 (183) [0.967]	36.2 ( 208) [0.381]
Former (n=99)	34.3 (34)	36.1 (36) [0.875]	29.3 (29) [0.672]
Current (n=449)	35.5 (376)	34.1 (153) [0.759]	30.4 (373) [0.138]
Consumption of alcohol, % (n)			
No (n=1090)	33.4 (364)	33.2 (362) [0.854]	33.4 (364) [1.000]
Yes (n=33)	36.4 (12)	30.3 (372) [0.651]	33.3 (375) [0.822]
Work‐related physical activity, % (n)			
Sedentary (n=451)	39.3 (177)	34.2 (154) [0.338]	26.6 (120) [0.025]
Moderate (n=354)	33.1 (117)	30.5 (108) [0.676]	36.6 (126) [0.567]
Vigorous (n=318)	33.5 (376)	33.1 (372) [0.908]	33.4 (375) [0.977]
Marital status, % (n)			
Unmarried ( n=41)	31.7 (13)	41.5 (17) [0.582]	26.8 ( 11) [0.793]
Married (n=1082)	33.5 (363)	32.8 (355) [0.842]	33.6 (364) [0.977]
Household wealth index, % (n)			
First quintile (n=223)	28.7 (64)	31.4 (70) [0.734]	39.9 (89) [0.153]
Second quintile (n=224)	32.1 (72)	34.8 (78) [0.726]	33.0 (74) [0.908]
Third quintile (n=220)	31.8 (70)	36.8 (81) [0.544]	31.4 (69) [0.960]
Fourth quintile (n=225)	36.4 (82)	30.7 (69) [0.461]	32.9 (74) [0.467]
Fifth quintile (n=224)	38.6 (87)	30.9 (367) [0.168]	30.4 (68) [0.288]
Reported hypertension diagnosis, % (n)			
No (n=171)	38.0 (65)	30.1 (53) [0.369]	30.1 (53) [0.369]
Yes (n=952)	32.7 (311)	33.5 (319) [0.873]	33.8 (322) [0.769]
Reported diabetes mellitus diagnosis, % (n)			
No (n=47)	23.4 (11)	34.0 (16) [0.554]	42.6 (20) [0.286]
Yes (n=1055)	33.7 (355)	33.3 (351) [0.910]	33.1 (349) [0.866]
Reported sleep hours, % (n)			
<6 h ( n=233)	37.8 (88)	32.6 (76) [0.487]	29.6 (69) [0.282]
6 to >9 h ( n=755)	33.4 (252)	32.7 (247) [0.868]	33.9 (256) [0.905]
≥9 h (n=135)	26.7 (36)	36.3 (49) [0.349]	37.0 (50) [0.315]
Participants consumption of table salt with food,^,^ % (n)			
No (n=727))	29.3 (213)	33.4 (243) [0.347]	37.3 (271) [0.065]
Yes (n=396)	41.2 (163)	32.1 (129) [0.585]	26.3 (104) [0.013]
Fasting blood glucose (mmol/L), mean (SD)	5.1 (1.6)	5 (1.3) [0.735]	5.5 (2.5) [0.003]
Serum cholesterol (mg/dL), mean (SD)	154.5 (43.9)	160.8 (42.7) [0.050]	157 (46.1) [0.449]
Serum triglycerides (mg/dL), mean (SD)	125.8 (96.9)	143.3 (115.4) [0.027]	161.6 (165.6)[0.004]
Serum HDL‐C (mg/dL), mean (SD)	37.3 (9.97)	36.5 (9.13) [0.252]	35.5 (9.95) [0.012]
Serum uric acid (mg/dL), mean (SD)	3.4 (1.7)	3.5 (1.7) [0.513]	3.6 (1.9) [0.151]
Urine protein (mg/dL), mean (SD)	155.6 (149.95)	181.1 (191.7) [0.047]	287.6 (1687.4) [0.139]

Tertile 1 of past urine Na, <142.81 mmol/day; tertile 2 of past urine Na, ≥142.81 to <182.36 mmol/day; and tertile 3 of past urine Na, ≥182.36 mmol/day. HDL‐C indicates high‐density lipoprotein cholesterol; and SD, standard deviation.

* *P* value for 2‐sample proportion or mean difference considering tertile 1 as the reference category.

We conducted 2 sensitivity analyses. An unhealthy diet such as processed and high carbohydrate–containing food is often associated with high Na intake, overweight conditions, and poor cardiometabolic health.^9^, ^33^ Therefore, our findings between Na intake and cardiometabolic biomarkers may be confounded by an unhealthy diet. We did not collect data on diet, which precluded our ability to adjust for it. Moreover, the effect of Na intake on cardiometabolic biomarkers can also be mediated through body fat deposition or weight gain.^9^ To avoid this possible bias by unhealthy diet, and to observe the direct association between Na intake and cardiometabolic biomarkers, we excluded the overweight and obese participants from analyses in the first sensitivity analyses based on Asian BMI categories (BMI, ≥23), and those with large waist circumference (≥92 cm for male and ≥89 cm for female). Evidence suggests increased urine Na excretion (natriuresis) among individuals with type 1 diabetes mellitus^34^ and urine Na retention among those with type 2 diabetes mellitus.^35^ Therefore, in a second sensitivity analysis, we assessed the relationship between past and contemporary Na exposures with fasting plasma glucose after excluding self‐reported diabetic participants. We only created the RCS plots for both sensitivity analyses. All statistical analyses were performed in Stata, version 15.0.

### Ethics Approval and Consent to Participate

Institutional review boards of the International Centre for Diarrheal Disease Research, Bangladesh (icddr.b) approved the study protocol. Informed written consent was obtained from all study participants.

## Results

The median age was 41 (interquartile range [IQR], 31–54) years, and the median BMI was 21.8 kg/m^2^ (IQR, 19.4–24.3 kg/m^2^). Of the participants, 41% were male, 30% were overweight, 40% were smokers, 3% reported alcohol consumption, and 40% had work‐related sedentary activities. The Pearson correlation coefficient between past and contemporary Na exposure was 0.54. Compared with participants of tertile 1 past Na exposure (urine Na, <142.81 mmol/day), tertile 3 (urine Na, >182.36 mmol/day) had a lower proportion of those ≥60 years old and sedentary and a higher proportion of overweight participants (Table 1). Participants with tertile 3 past Na exposure had a higher fasting blood glucose, triglycerides, and lower HDL‐C compared with tertile 1 participants (Table 1).

**Figure 2:**
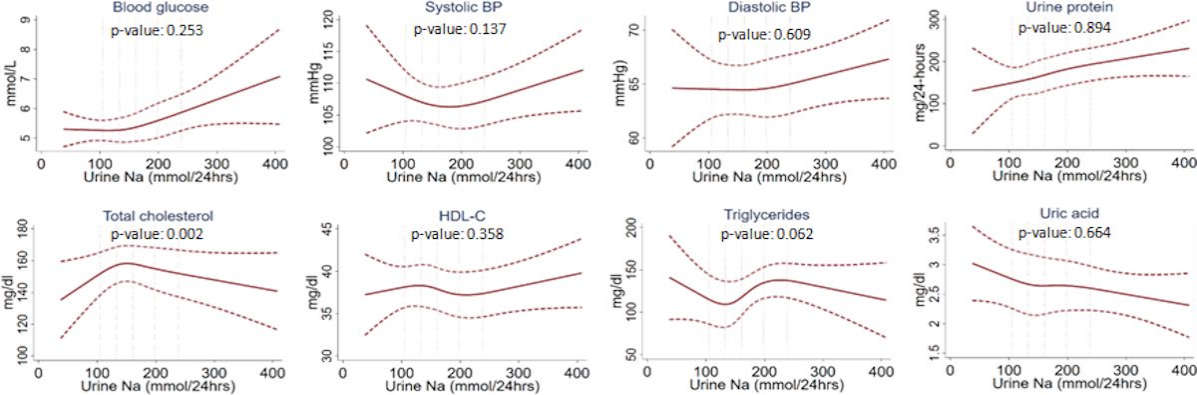
Restricted cubic spline plots (solid lines) and 95% CI (dashed lines) for past Na exposure and cardiometabolic biomarker relationships when adjusted for age, sex, BMI, smoking, physical activities, alcohol consumption, sleep hours, religion, and household wealth. The 5 vertical lines indicate the 10th, 25th, 50th, 75th, and 90th percentiles of 24UNa distribution. *P*<0.05 indicates departure from linearity. The relationship between past Na intake and cardiometabolic biomarkers is linear, except for total cholesterol. 24UNa indicates 24‐hour urine Na; BMI, body mass index; BP, blood pressure; and HDL‐C, high‐density lipoprotein cholesterol.

**Figure 3:**
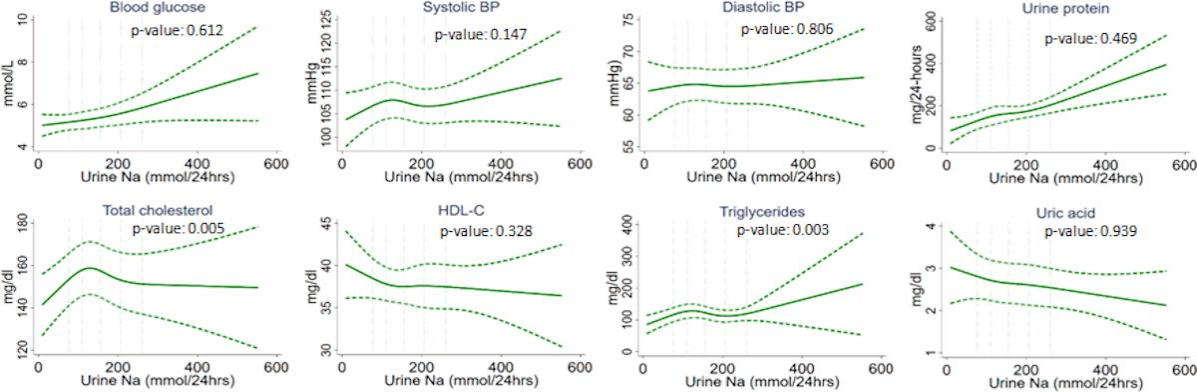
Restricted cubic spline plots (solid lines) and 95% CI (dashed lines) for contemporary Na exposure and cardiometabolic biomarker relationships when adjusted for age, sex, BMI, smoking, physical activities, alcohol consumption, sleep hours, religion, and household wealth. The 5 vertical lines indicate the 10th, 25th, 50th, 75th, and 90th percentiles of 24UNa distribution. *P*<0.05 indicates departure from linearity. 24UNa indicates 24‐hour urine Na; BMI, body mass index; BP, blood pressure; and HDL‐C, high‐density lipoprotein cholesterol.

For both past and contemporary Na exposure, RCS plots illustrated a positive linear relationship with 24UNa and fasting blood glucose and urine total protein, a negative linear association with 24UNa and uric acid, and an inverse U‐shaped relationship with 24UNa and total cholesterol (Figures 2 and 3). For systolic BP, a U‐shaped relationship was observed for past Na exposure but a positive linear relationship for contemporary Na (Figures 2 and 3).

In the fully adjusted model, compared with participants in tertile 1 of past Na (urine Na, <142.81 mmol/day), those in tertile 3 (urine Na, >182.36 mmol/day) had 0.44 mmol/L (95% CI, 0.19–0.70 mmol/L) higher fasting glucose and 1.19 (95% CI, 1.03–1.38) ratio of median urine protein (Table 2). Similarly, compared with participants in tertile 1 of contemporary Na (urine Na <125.07 mmol/day), those in tertile 3 (urine Na, >186.46 mmol/day) had 0.56 mmol/L (95% CI, 0.17–0.94 mmol/L) higher fasting glucose and 1.40 (95% CI, 1.22–1.61) ratio of median urine protein (Table 2). We did not find any associations between past or contemporary Na categories with systolic BP, total cholesterol, triglycerides, HDL‐C, and uric acid in linear models when Na was used as a continuous exposure.

**Table 2:** The Differences in Cardiometabolic Biomarkers Associated Tertile 2 and 3 Urine Na Compared With Tertile 1, Both for Past and for Contemporary Na Exposure

Biomarkers	Past Na Exposure			Contemporary Na Exposure		
	Tertile 1	Tertile 2, β (95% CI)	Tertile 3, β (95% CI)	Tertile 1	Tertile 2, β (95% CI)	Tertile 3, β (95% CI)
Blood glucose, mmol/L						
Model 1	Ref	−0.29 (−0.20 to 0.14)	0.50 (0.23 to 0.78)	Ref	−0.01 (−0.30 to 0.29)	0.61 (0.25 to 0.98)
Model 2	Ref	−0.03 (−0.20 to 0.14)	0.44 (0.12 to 0.77)	Ref	0.02 (−0.25 to 0.30)	0.57 (0.17 to 0.96)
Model 3	Ref	−0.02 (−0.17 to 0.13)	0.44 (0.19 to 0.70)	Ref	0.04 (−0.24 to 0.31)	0.56 (0.17 to 0.94)
Urine total protein,† mg/dL						
Model 1	Ref	1.13 (1.03 to 1.25)	1.29 (1.16 to 1.42)	Ref	1.21 (1.07 to 1.37)	1.45 (1.27 to 1.66)
Model 2	Ref	1.12 (0.99 to 1.26)	1.21 (1.04 to 1.40)	Ref	1.16 (1.00 to 1.35)	1.41 (1.21 to 1.63)
Model 3	Ref	1.11 (0.99 to 1.24)	1.19 (1.03 to 1.38)	Ref	1.16 (1.04 to 1.35)	1.40 (1.22 to 1.61)
Systolic BP to mm Hg						
Model 1	Ref	−1.01 (−2.93 to 0.91)	−1.27 (−3.10 to 0.54)	Ref	−0.64 (−3.00 to 1.73)	−0.55 (−2.66 to 1.56)
Model 2	Ref	−0.70 (−2.07 to 0.67)	−1.55 (−3.51 to 0.40)	Ref	0.37 (−1.40 to 2.14)	−0.46 (−2.42 to 1.50)
Model 3	Ref	−0.66 (−2.00 to 0.68)	−1.18 (−3.17 to 0.81)	Ref	0.46 (−1.30 to 1.21)	−0.25 (−2.07 to 1.58)
Diastolic BP, mm Hg						
Model 1	Ref	0.35 (−1.38 to 2.08)	0.84 (−0.21 to 1.88)	Ref	−0.76 (−2.51 to 1.00)	−0.01 (−1.49 to 1.48)
Model 2	Ref	0.05 (−1.18 to 1.28))	−0.32 (−1.42 to 0.77)	Ref	−0.18 (−1.55 to 1.19)	−0.37 (−1.75 to 1.00)
Model 3	Ref	0.12 (−1.11 to 1.35)	−0.02 (−1.08 to 1.03)	Ref	−0.10 (−1.45 to 1.25)	−0.18 (−1.57 to 1.21)
Total cholesterol, mg/dL						
Model 1	Ref	8.27 (2.67 to 13.88)	2.40 (−4.93 to 9.74)	Ref	−1.68 (−6.64 to 3.29)	−2.66 (−7.77 to 2.46)
Model 2	Ref	8.39 (3.73 to 13.05)	0.59 (−5.76 to 6.94)	Ref	0.29 (−5.62 to 6.21)	−4.12 (−8.82 to 0.57)
Model 3	Ref	8.89 (4.01 to 13.76)	0.58 (−6.67 to 7.82)	Ref	0.61 (−5.54 to 6.76)	−4.07 (−8.80 to 0.66)
HDL‐C, mg/dL						
Model 1	Ref	−057 (−2.35 to 1.22)	−1.59 (−2.82 to −0.35)	Ref	0.18 (−1.07 to 1.43)	−0.69 (−1.88 to 0.50)
Model 2	Ref	−0.09 (−1.97 to 1.80)	−0.59 (−1.95 to 0.76)	Ref	0.00 (−1.31 to 1.31)	−0.43 (−1.44 to 0.58)
Model 3	Ref	0.05 (−1.87 to 1.97)	−0.60 (−2.03 to 0.83)	Ref	0.07 (−1.18 to 1.32)	−0.36 (−1.45 to 0.74)
Triglycerides to mg/dL						
Model 1	Ref	12.58 (−0.45 to 25.60)	30.24 (8.46 to 52.02)	Ref	−14.02 (−24.85 to −3.19)	−2.88 (−16.67 to 10.90)
Model 2	Ref	9.31 (−2.01 to 20.64)	21.64 (1.07 to 42.21)	Ref	−7.64 (−18.01 to 2.74)	−2.43 (−16.23 to 11.37)
Model 3	Ref	8.97 (−2.06 to 19.99)	22.23 (0.23 to 44.23)	Ref	−6.95 (−17.69 to 3.79)	−1.20 (−16.51 to 14.11)
Uric acid, mg/dL						
Model 1	Ref	0.04 (−0.18 to 0.26)	0.07 (−0.19 to 0.33)	Ref	−0.19 (−0.41 to 0.03)	−0.26 (−0.43 to −0.09)
Model 2	Ref	−0.03 (−0.18 to 0.13)	−0.05 (−0.27 to 0.17)	Ref	−0.10 (−0.33 to −0.13)	−0.20 (−0.38 to −0.03)
Model 3	Ref	−0.01 (−0.17 to 0.15)	−0.01 (−0.24 to 0.23)	Ref	−0.78 (−0.32 to 0.16)	−0.17 (−0.37 to 0.02)

Tertile 1 of past urine Na, <142.81 mmol/day; tertile 2 of past urine Na, ≥142.81 to <182.36 mmol/day; and tertile 3 of past urine Na, ≥182.36 mmol/day. Tertile 1 of contemporary urine Na, <125.07 mmol/day; tertile 2 of contemporary urine Na, ≥142.81 to <186.46 mmol/day; and tertile 3 of contemporary urine Na, ≥186.46 mmol/day. Model 1, unadjusted; model 2, adjusted for age, sex, and BMI; model 3, adjusted for age, sex, BMI, smoking, use of alcohol, physical activity, marital status, sleep hours, consumption of table salt with food, and household wealth. BMI indicates body mass index; and HDL‐C, high‐density lipoprotein cholesterol.

* β denotes difference in mean concentrations of cardiometabolic biomarkers compared with the reference group (tertile 1).

† For urine total protein, β refers to ratio of median urine protein where reference group (tertile 1) is the denominator.

Compared with participants in tertile 1 of past Na (urine Na, <142.81 mmol/day), those in tertile 3 (urine Na, >182.36 mmol/day) had 1.46 (95% CI, 1.08–1.99) times higher prevalence of prediabetes or diabetes mellitus, 5.49 (95% CI, 2.73–11.01) times higher prevalence of large waist circumference, and 1.60 (95% CI, 1.04–2.46) times higher prevalence of metabolic syndrome (Table 3). Compared with participants in tertile 1 of contemporary Na (urine Na, <125.07 mmol/day), those in tertile 3 (urine Na, >186.46 mmol/day) had 1.93 (95% CI, 1.24–3.00) times higher prevalence of prediabetes or diabetes mellitus, 3.14 (95% CI, 1.45–6.83) times higher prevalence of proteinuria, and 2.23 (95% CI, 1.34–3.71) times higher prevalence of large waist circumference (Table 4). We did not find any associations between past or contemporary Na tertiles with elevated plasma triglycerides or cholesterol or uric acid and reduced HDL‐C.

**Table 3:** Prevalence Ratios for Elevated Cardiometabolic Biomarkers and Metabolic Syndrome Among Tertile 2 and 3 Participants of Past Na Exposure Compared With Tertile 1

24UNa	Unadjusted β* (95% CI)	Adjusted for Age, Sex, and BMI β* (95% CI)	Multivariable‐Adjusted^†^ β* (95% CI)
Prediabetes or diabetes mellitus (≥5.5 mmol/L; 24%)			
Tertile 1	Referent	Referent	Referent
Tertile 2	0.85 (0.62–1.16)	0.76. (0.55–1.05)	0.79 (0.56–1.11)
Tertile 3	1.71 (1.20–2.44)	1.36 (0.98–1.91)	1.46 (1.08–1.99)
Elevated plasma total cholesterol (≥200 mg/dL; 16%)			
Tertile 1	Referent	Referent	Referent
Tertile 2	1.35 (0.80–2.28)	1.30 (0.77–2.22)	1.36 (0.79–2.33)
Tertile 3	0.99 (0.59–1.65)	0.85 (0.49–1.48)	0.87 (0.49–2.53)
Reduced plasma HDL‐C (<40 mg/dL for male; <50 mg/dL for female; 18%)			
Tertile 1	Referent	Referent	Referent
Tertile 2	1.08 (0.72–1.63)	1.00 (0.59–1.70)	0.94 (0.54–1.63)
Tertile 3	1.36 (0.82–2.26)	1.00 (0.54–1.84)	0.92 (0.48–1.74)
Elevated plasma triglycerides (≥150 mg/dL; 33%)			
Tertile 1	Referent	Referent	Referent
Tertile 2	1.21 (0.89–1.64)	1.12 (0.82–1.55)	1.07 (0.77–1.50)
Tertile 3	1.48 (1.04–2.12)	1.23 (0.85–1.77)	1.21 (0.80–1.83)
Elevated plasma uric acid (≥7 mg/dL for male; ≥6 mg/dL for female; 5%)			
Tertile 1	Referent	Referent	Referent
Tertile 2	0.90 (0.47–1.71)	0.75 (0.41–1.39)	0.83 (0.43–1.58)
Tertile 3	1.02 (0.57–1.83)	0.73 (0.41–1.31)	0.79 (0.42–1.47)
Proteinuria (≥300 mg/dL; 15%)			
Tertile 1	Referent	Referent	Referent
Tertile 2	1.73 (1.01–2.95)	1.62 (0.89–2.92)	1.66 (0.88–3.11)
Tertile 3	2.26 (1.32–3.85)	1.81 (0.94–3.45)	1.68 (0.85–3.31)
Large waist circumference (≥92 cm for male or ≥89 cm for female)			
Tertile 1	Referent	Referent	Referent
Tertile 2	1.53 (0.92–2.56)	1.76 (0.90–3.43)	2.12 (1.19–3.78)
Tertile 3	3.61 (1.92–6.77)	4.38 (2.00–9.60)	5.49 (2.73–11.01)
Metabolic syndrome			
Tertile 1	Referent	Referent	Referent
Tertile 2	1.09 (0.70–1.72)	0.97 (0.57, 1.65)	0.99 (0.61, 1.61)
Tertile 3	2.05 (1.31, 3.20)	1.45 (0.93, 2.25)	1.60 (1.04, 2.46)

Tertile 1 of past urine Na, <142.81 mmol/day; tertile 2 of past urine Na, ≥142.81 to <182.36 mmol/day; and tertile 3 of past urine Na, ≥182.36 mmol/day. 24UNa indicates 24‐hour urine Na; and HDL‐C, high‐density lipoprotein cholesterol.

* β denotes prevalence ratio where reference group (tertile 1) is the denominator.

† Adjusted for age, sex, BMI, smoking, alcohol, physical activity, marital status, religion, sleep hours, consumption of table salt with food, and household wealth.

**Table 4:** Prevalence Ratios for Elevated Cardiometabolic Biomarkers and Metabolic Syndrome Among Tertile 2 and 3 Participants of Contemporary Na Exposure Compared With Tertile 1

24UNa	Unadjusted β* (95% CI)	Adjusted for Age, Sex and BMI β* (95% CI)	Multivariable‐Adjusted^†^ β* (95% CI)
Prediabetes or diabetes mellitus (≥5.5 mmol/L; 24%)			
Tertile 1	Referent	Referent	Referent
Tertile 2	1.07 (0.59–1.92)	1.14 (0.64–2.02)	1.16 (0.65–2.07)
Tertile 3	2.02 (1.36–3.01)	1.88 (1.21–2.93)	1.93 (1.24–3.00)
Elevated plasma total cholesterol (≥200 mg/dL; 16%)			
Tertile 1	Referent	Referent	Referent
Tertile 2	0.8 (0.51–1.33)	0.92 (0.53–1.59)	0.92 (0.54–1.59)
Tertile 3	0.97 (0.71–1.33)	0.94 (0.68–1.29)	0.92 (0.65–1.28)
Reduced plasma HDL‐C (<40 mg/dL for male; <50 mg/dL for female; 18%)			
Tertile 1	Referent	Referent	Referent
Tertile 2	1.02 (0.67–1.57)	0.97 (0.63–1.50)	0.97 (0.62–1.52)
Tertile 3	1.41 (1.03–1.93)	1.18 (0.80–1.75)	1.13 (0.75–1.69)
Elevated plasma triglycerides (≥150 mg/dL; 33%)			
Tertile 1	Referent	Referent	Referent
Tertile 2	0.64 (0.48–0.86)	0.66 (0.47–0.93)	0.66 (0.46–0.93)
Tertile 3	0.86 (0.71–1.05)	0.80 (0.63–1.01)	0.80 (0.59–1.07)
Elevated plasma uric acid (≥7 mg/dL for male; ≥6 mg/dL for female; 5%)			
Tertile 1	Referent	Referent	Referent
Tertile 2	0.43 (0.22–0.83)	0.43 (0.21–0.88)	0.43 (0.21–0.86)
Tertile 3	0.73 (0.36–1.48)	0.62 (0.28–1.33)	0.61 (0.28–1.30)
Proteinuria (≥300 mg/dL; 15%)			
Tertile 1	Referent	Referent	Referent
Tertile 2	1.51 (0.74–3.06)	1.42 (0.68–2.96)	1.38 (0.67–2.84)
Tertile 3	3.74 (1.72–8.12)	3.25 (1.48–7.1)	3.14 (1.45–6.83)
Large waist circumference (≥92 cm for male or ≥89 cm for female)			
Tertile 1	Referent	Referent	Referent
Tertile 2	1.07 (0.50–2.29)	1.08 (0.47–2.55)	1.24 (0.55–2.79)
Tertile 3	1.91 (1.14–3.19)	1.84 (1.04–3.25)	2.23 (1.34–3.71)
Metabolic syndrome			
Tertile 1	Referent	Referent	Referent
Tertile 2	0.79 (0.45–1.38)	0.89 (0.50–1.59)	0.90 (0.52–1.54)
Tertile 3	1.53 (1.09–2.17)	1.28 (0.82–1.97)	1.36 (0.89–2.07)

Tertile 1 of contemporary urine Na, <125.07 mmol/day, tertile 2 of contemporary urine Na, ≥142.81 to <186.46 mmol/day, and tertile 3 of contemporary urine Na, ≥186.46 mmol/day. 24UNa indicates 24‐hour urine Na; and HDL‐C, high‐density lipoprotein cholesterol.

* β denotes prevalence ratio where reference group (tertile 1) is the denominator.

† Adjusted for age, sex, BMI, smoking, alcohol, physical activity, marital status, religion, sleep hours, consumption of table salt with food, and household wealth.

### Sensitivity Analyses

When the overweight, obese, and large waist circumference participants were excluded in the first sensitivity analysis, the relationships between urine Na and cardiometabolic biomarkers in RCS plots remained unchanged except for the loss of U‐shape associations for triglycerides (Figure S2). The linear positive association between the 24UNa and fasting blood glucose in RCS plots remained similar when person‐visits of self‐reported diabetic participants were excluded from analyses (Figure S3).

## Discussion

Our analyses suggest that both past and contemporary high Na intake had a linear positive association with fasting blood glucose and urine total protein. We also found that both past and contemporary Na intake was associated with higher prevalence of prediabetes or diabetes mellitus, proteinuria, and large waist circumference. Past Na intake was additionally associated with higher prevalence of metabolic syndrome. The magnitudes of association for large waist circumference and metabolic syndrome were stronger for past Na intake, whereas magnitudes of association for prediabetes or diabetes mellitus and proteinuria were stronger for contemporary Na.

Salt or Na has no calories, but as highlighted by our findings, several molecular mechanisms also suggest that Na intake can increase the risk of diabetes mellitus, obesity, and metabolic syndrome. High Na intake regulates the glucose and fructose metabolism and induces insulin and leptin resistance.^36^, ^37^, ^38^ The Bangladeshi population has an increasing prevalence of type 2 diabetes mellitus and metabolic syndrome,^39^, ^40^ and to our best knowledge, this is the first study that explored the association between high Na intake and fasting blood glucose and metabolic syndrome among this population. Studies in other settings also suggest the association between high Na intake and incidence of diabetes mellitus,^41^, ^42^ markers of insulin resistance,^43^ or abdominal obesity.^44^ An unhealthy or poor diet rich in carbohydrate or fat may confound our findings since excessive Na is often ingested along with the poor diet^45^; however, the positive association between urine Na and fasting plasma glucose persisted even after excluding the overweight or obese participants in sensitivity analyses. All our RCS plots also suggest past and contemporary Na exposure were associated with urine total protein in a monotonic way. High Na intake increases the vascular endothelial dysfunction and microvasculature permeability and causes subsequent leakage of protein from the vasculature.^46^, ^47^, ^48^ Proteinuria is an independent biomarker for future cardiovascular diseases risk^49^, ^50^, ^51^, ^52^ and is associated with the pathogenesis of hypertension,^53^, ^54^ chronic kidney disease,^55^ myocardial ischemia,^56^ carotid artery thickness,^57^, ^58^ and left ventricular hypertrophy.^59^, ^60^

Our RCS plots suggest lower 24UNa intake was associated with higher concentrations of uric acid. The relationship between Na intake and blood uric acid is controversial. Epidemiologic studies suggest high Na intake is associated with an increased level of blood uric acid,^61^, ^62^ but studies also noted the opposite relationship due to renal clearance of uric acid.^63^, ^64^ The U‐shaped RCS plot for triglycerides suggests that past Na intake may be associated with high triglycerides levels, but such U‐shaped association was lost for contemporary Na exposure. A systematic review demonstrated that Na restricted diet increases the blood triglycerides levels by 6.3%,^65^ but the Dietary Approaches to Stop Hypertension (DASH) multicenter randomized trial did not find any association between Na intake and triglycerides.^66^

We found an inverse‐U shaped association between urine Na and total cholesterol in RCS plots, but linear and tertile models did not demonstrate a significant difference at 5% level of significance: the same was true for HDL‐C. A contemporary analysis from the Korea National Health and Nutrition Examination Survey IV‐V databases (2008–2011) that encompassed 18 146 adults' data suggested a negative association between urine Na and HDL‐C (*P*≤0.001),^42^ but the daily urine Na levels in that survey were estimated from fasting morning samples. The DASH multicenter randomized trial did not find any association between Na intake and serum total cholesterol or HDL‐C.^66^ Nevertheless, a systematic review demonstrated that Na‐restricted diet increase the blood cholesterol levels by 2.9%.^65^

We did not find any statistical relationship between past or contemporary Na intake with BP. Nevertheless, we have reported a positive statistically significant association between 24‐hour Na intake and BP in the same study population elsewhere.^21^, ^67^ Several factors may have contributed to such altered Na intake and BP association in current analyses. First, BP data used in this article were measured in the fifth visit of the stepped‐wedge trial (April 2017), which was hot summer in Bangladesh. Epidemiologic studies suggest that ambient temperature influences BP and lower mean population BP more during the summer than during the cold months, which is due to temperature‐induced dilatation of the skin vasculature that lowers BP.^68^, ^69^, ^70^ Second, past Na intake may not be appropriate exposure for BP since salt intake associated hemodynamic changes that influence BP start immediately after salt intake.

Our study has several important limitations. We only had single measurements of cardiometabolic biomarkers, which may be affected by several factors such as food intake of the previous day, stress and anxiety level, or the duration of sleep attained on the previous night.^71^, ^72^ Therefore, a contemporary measurement of biomarkers may not be reflective of actual disease risk.^73^ Our 24‐hour urine sample collections from the participants at the population level were likely affected by over‐ and undercollection.^74^ Twenty‐four‐hour urine collection studies are recommended to incorporate estimation of completeness of 24‐hour urine using para‐aminobenzoic acid,^75^ a gold standard approach of determining completeness. Having a lack of that component, we are unable to evaluate the actual bias associated with the incomplete collection of 24‐hour urine samples. We found high correlation between past and contemporary Na exposures, which explains near‐similar relationship of past and contemporary Na exposures with cardiometabolic biomarkers. Collecting data on disease incidence through longitudinal follow‐up visits will better capture the relationship between Na concentrations and cardiometabolic disease risk. However, such longitudinal data are expensive to gather in low‐income settings where routine disease surveillance is generally absent.

Our findings provide evidence that both past and contemporary Na intake is strongly associated with a higher prevalence of prediabetes or diabetes mellitus, proteinuria, abdominal obesity, and metabolic syndrome. These findings suggest high‐Na‐containing diet may increase the cardiometabolic disease risks of the population.

## Sources of Funding

This research was funded by Wellcome Trust, UK, Our Planet, Our Health Award (Grant 106871/Z/15/Z). Dr Gribble's effort was supported in part by funding from the National Institute of Environmental Health Sciences (P30 ES019776).

## Disclosures

None.

## Supporting information


**Figures S1–S3**
