# Supplementary material for: Past Sodium Intake, Contemporary Sodium Intake, and Cardiometabolic Health in Southwest Coastal Bangladesh
Source: J Am Heart Assoc. 2020 Sep 2;9(18):e014978. doi: 10.1161/JAHA.119.014978 (PMC7727005; doi:10.1161/JAHA.119.014978)
Supplement: Supplementary file 1 — Figures S1–S3 [file JAH3-9-e014978-s001.pdf]

SUPPLEMENTAL MATERIAL

Figure S1. Working hypothesis on how sodium intake is linked to type 2 diabetes and insulin sensitivity.

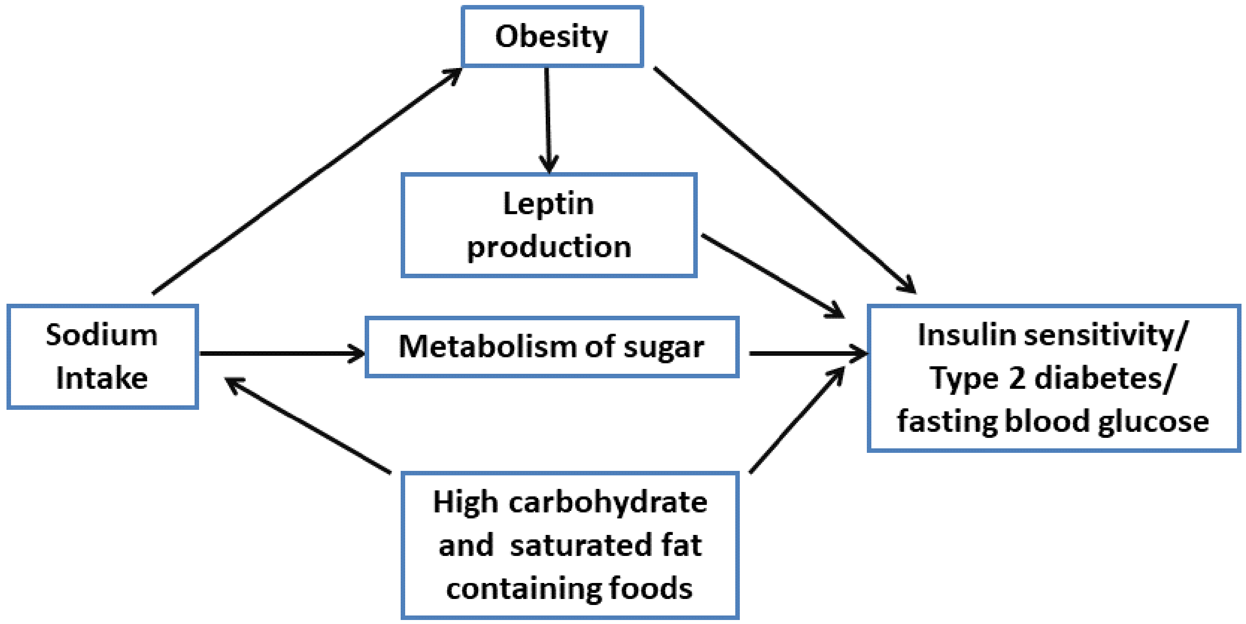

Figure S2. Sensitivity analyses—restricted cubic spline plots (solid lines) and 95% confidence interval (dashed lines) for past versus contemporary Na exposure and cardiometabolic biomarker relationships when the overweight and obese participants and those with large waist circumference were excluded from analyses.

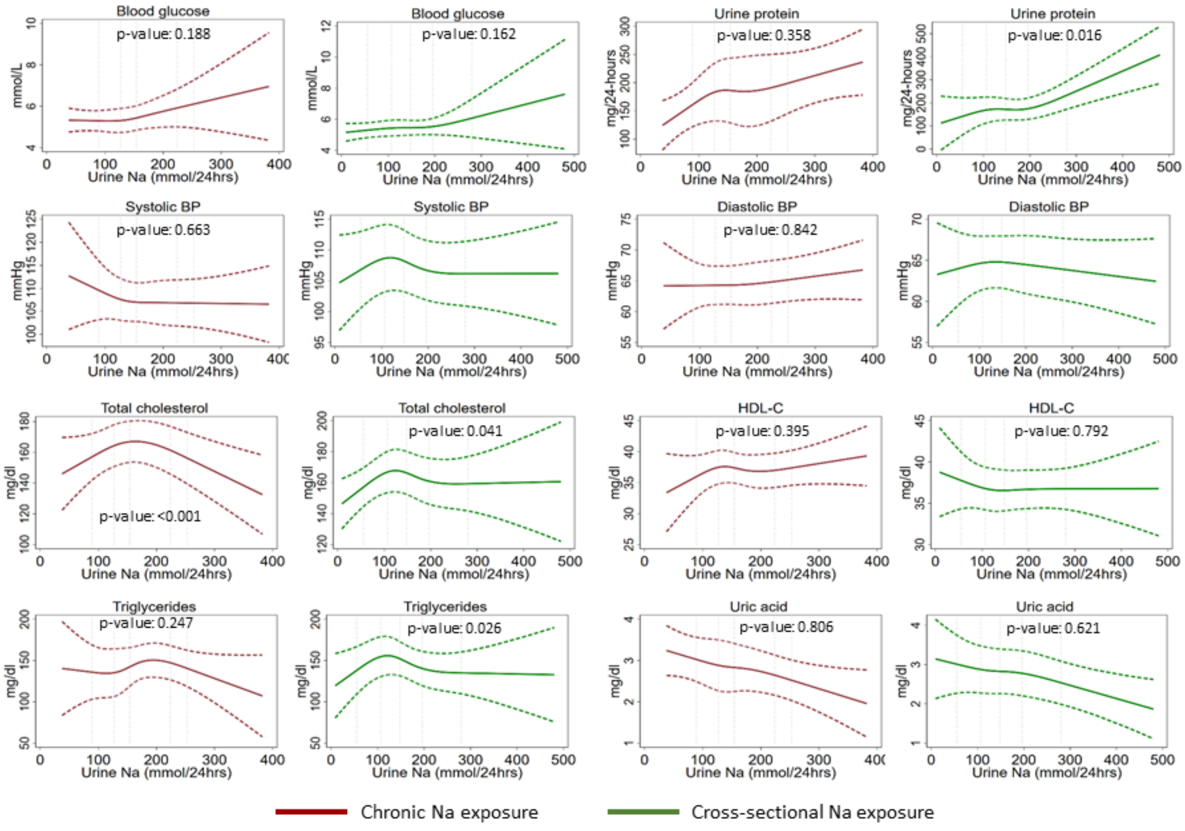

Plots were adjusted for age, sex, smoking, physical activities, alcohol consumption, sleep hour, religion and household wealth. The five vertical lines indicate the 10th, 25th, 50th, 75th and 90th percentiles of 24-hour urine Na distribution.

Figure S3. Sensitivity analyses—restricted cubic spline plot (solid line) and 95% confidence interval (dashed lines) for past versus contemporary Na exposure and fasting blood glucose relationship when the analyses were restricted among non-diabetic participants.

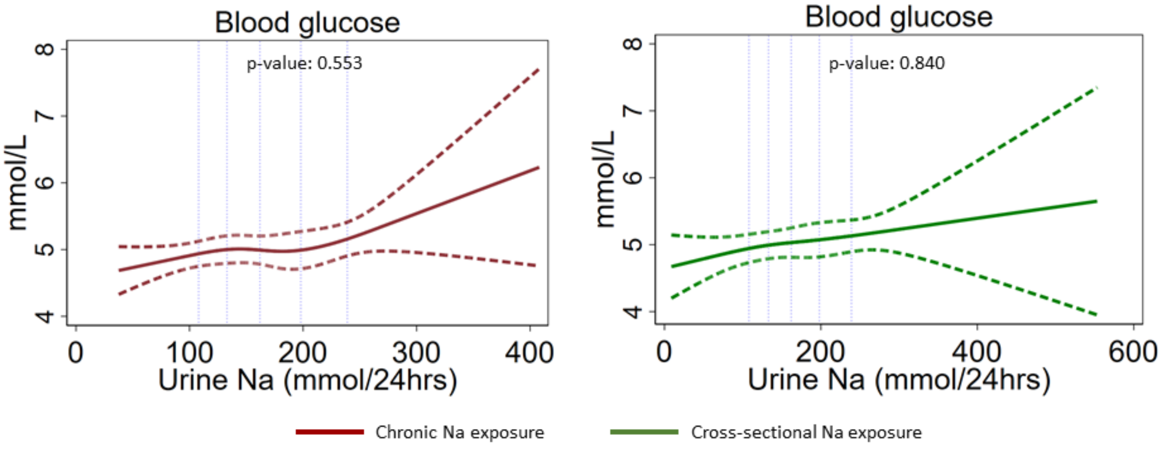

Plots were adjusted for age, sex, BMI, smoking, physical activities, alcohol consumption, sleep hour, religion and household wealth. The five vertical lines indicate the 10th, 25th, 50th, 75th and 90th percentiles of 24-hour urine Na distribution.
